# Young Investigator Award 2026: Integrative mechanistic insights into YAP/TAZ-mediated angiogenesis and its therapeutic implications

**DOI:** 10.1007/s00210-026-05654-6

**Published:** 2026-07-02

**Authors:** Yohanes Cakrapradipta Wibowo

**Affiliations:** 1 Experimental Pharmacology Mannheim, European Center for Angioscience (ECAS), Mannheim Medical Faculty, Heidelberg University, Mannheim, Germany; 2 Department of Cardiology, Angiology, Haemostaseology and Medical Intensive Care, European Center for Angioscience (ECAS), Medical Faculty Mannheim, University Medical Centre Mannheim, Heidelberg University, Mannheim, Germany; 3 Cardiovascular Systems Biology, Medical Centre Mannheim, Medical Faculty Mannheim, Heidelberg University, 68167 Mannheim, Germany; 4 Helmholtz-Institute for Translational AngioCardioScience (HI-TAC), Max Delbrück Center for Molecular Medicine in Helmholtz Association (MDC) at Heidelberg University, 69117 Heidelberg, Germany; 5 German Centre for Cardiovascular Research (DZHK), Partner site Heidelberg/Mannheim, Heidelberg/Mannheim, Germany

**Keywords:** YAP/TAZ, Angiogenesis, Endothelial cells, Tankyrases

## Abstract

The Young Investigator Award or “Nachwuchspreis der DGPT” (formerly the Fritz Külz Award) is awarded by the German Society for Experimental and Clinical Pharmacology and Toxicology (Deutsche Gesellschaft für experimentelle und klinische Pharmakologie und Toxikologie e.V./DGPT) to young scientists in recognition of their outstanding work in experimental pharmacology and toxicology. The Young Investigator Award in 2026 was awarded to Dr. Dr. Yohanes Cakrapradipta Wibowo from the Medical Faculty Mannheim of Heidelberg University for his previous PhD research, which was supervised by the late Prof. Dr. Thomas Wieland. In this work, Dr. Wibowo uncovered a new mechanistic insight into angiogenesis and proposed a therapeutic strategy for treating pathological angiogenesis. This article provides an overview of Dr. Wibowo’s research.

Angiogenesis is the essential biological process by which new blood vessels are formed from preexisting vascular structures. This process plays an important role in both physiological and pathological conditions (Carmeliet 2003; Wacker and Gerhardt 2011). Proper angiogenesis is mandatory for numerous processes from wound healing, tissue development, and angiogenesis-related disease progression such as cancers (Carmeliet and Jain 2000).

Over the past few decades, considerable efforts have been devoted to investigating the biological mechanisms that modulate angiogenesis. Among those, vascular endothelial growth factor/vascular endothelial growth factor receptor 2 (VEGF/VEGFR2) and Notch signaling pathway have been proposed to be crucial regulators of angiogenesis. The balance between VEGF/VEGFR2 and Notch signaling allows tip and stalk cell specification [reviewed in (Blanco and Gerhardt 2013)].

Among VEGFR2 downstream targets, yes-associated protein/transcriptional coactivator with PDZ-binding motif (YAP/TAZ) has emerged as one of the most crucial effectors in angiogenesis. YAP/TAZ is a co-transcriptional activator that mediates gene expression through interaction via DNA-binding transcription factors, particularly transcriptional enhanced associate domains (TEADs) and is controlled by Hippo pathway (Elaimy and Mercurio 2018). Their involvement in the endothelial function and angiogenesis has been documented across embryonic development (Wang et al. 2017) and in both physiological (Nakajima et al. 2017) and pathological angiogenesis (Wang et al. 2016; Kim et al. 2017; Shen et al. 2021).

Due to its significant role in angiogenesis, YAP/TAZ has attracted wider interest as one of the most potential therapeutic targets in angiogenesis-related diseases. In his work, Dr. Wibowo has recently described that a tankyrase inhibitor, XAV939, exhibits a YAP/TAZ inhibitory property through the regulation of angiomotin-like 1 and 2 (AMOTL1/L2) (Ma et al. 2024). He further demonstrated that XAV939 treatment reduced YAP/TAZ expression levels and, more importantly, disrupted angiogenic signaling by inhibiting VEGFR2 trafficking. The disruption on VEGFR2 trafficking might be a negative feedback as a result of YAP/TAZ inhibition that has already previously been reported by Wang et al. (2017).

Dr. Wibowo further expanded his study on YAP/TAZ and found that it is also indirectly controlled by cyclic adenosine monophosphate (cAMP) signaling. In a paper currently under revision, using a pathological angiogenesis mice model, he observed that cAMP signaling regulates YAP/TAZ through junctional remodeling which maintained by a mechanosensory complex consists of vascular endothelial cadherin (VE-cadherin), VEGFR2, and platelet endothelial cell adhesion molecule-1 (PECAM1) (Fig. 1).

**Fig. 1:**
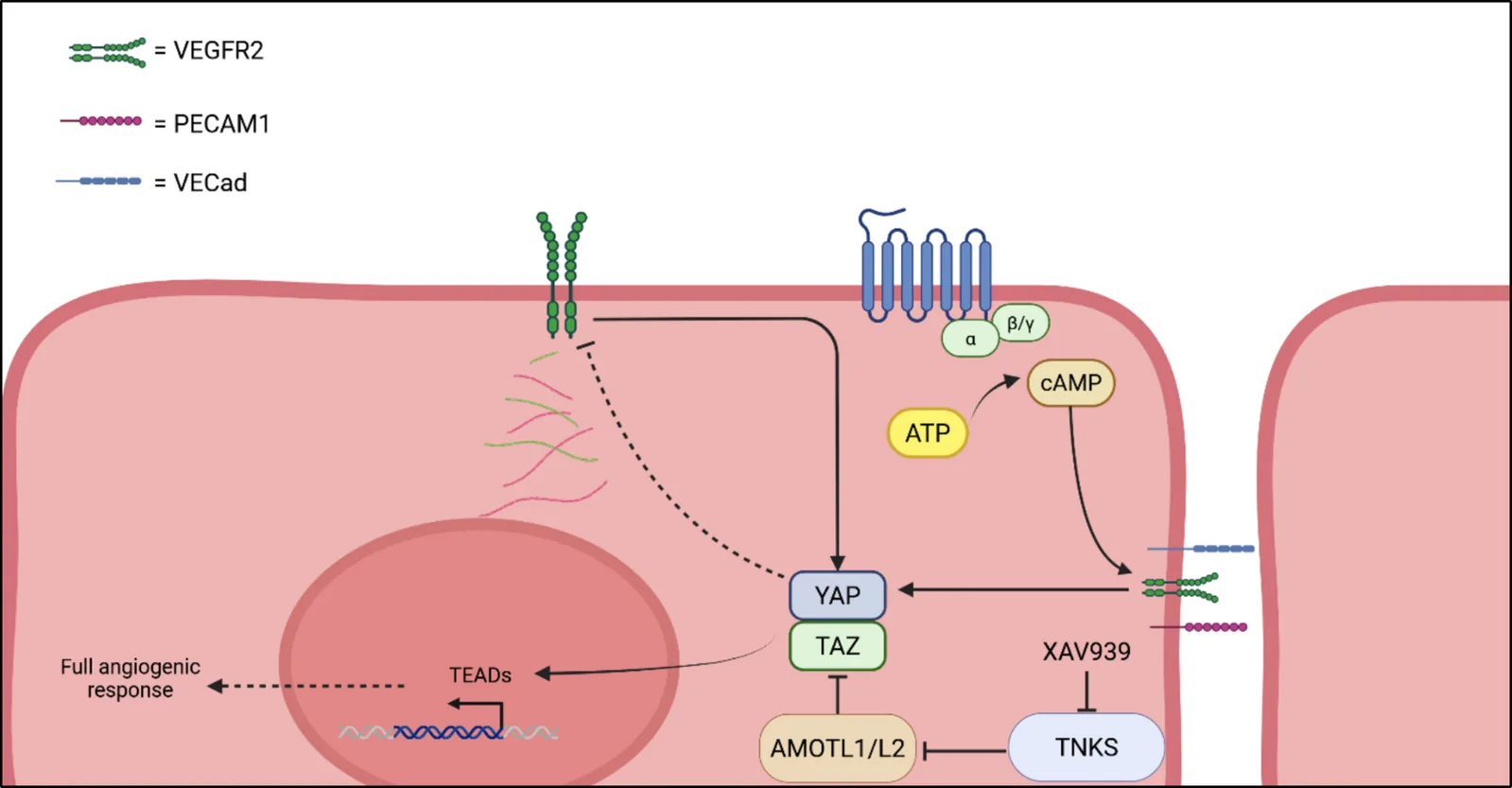
Endothelial YAP/TAZ is regulated by multiple stimuli. In endothelial cells, YAP/TAZ acts downstream of VEGFR2. cAMP, for example, via GPCR-dependent signaling, may also indirectly promote YAP/TAZ activation through a mechanosensory complex consists of VE-cadherin, VEGFR2, and PECAM1. In contrast, XAV939, a tankyrase (TNKS) inhibitor, inhibits YAP/TAZ activation through AMOTL1/2 regulation

To sum up, YAP/TAZ is a critical regulator in angiogenesis which can be activated by multiple stimuli such as VEGFR2 or indirectly by cAMP signaling. Targeting YAP/TAZ offers a new strategy to target angiogenesis in patients but special attention needs to be considered. Targeting YAP/TAZ may result in similar side effects to anti-VEGF therapy, as it also disrupts junctions and can potentially cause bleeding (Wang et al. 2017; Ma et al. 2024).
